# Special Issue “*Sporothrix* and Sporotrichosis 2.0”

**DOI:** 10.3390/jof8080821

**Published:** 2022-08-05

**Authors:** Héctor M. Mora-Montes

**Affiliations:** Departamento de Biología, División de Ciencias Naturales y Exactas, Campus Guanajuato, Universidad de Guanajuato, Noria Alta s/n, col. Noria Alta, C.P., Guanajuato 36050, Gto., Mexico; hmora@ugto.mx; Tel.: +52-473-732-0006 (ext. 8193)

**Keywords:** fungal pathogen, antifungal, immune system, diagnosis, virulence factor, adhesins, *Sporothrix schenckii*, feline sporotrichosis, *Sporothrix brasiliensis*, *Sporothrix globosa*

Sporotrichosis is a chronic fungal disease of humans and other mammals that often affects the skin and subcutaneous tissues and, rarely, deep-seated organs (most frequently in immunocompetent hosts) [1]. Although the etiological agents, which are members of the *Sporothrix* pathogenic clade, are distributed worldwide, the major toll of this disease is concentrated in tropical and subtropical regions [2]. The first fungal species associated with the disease was *Sporothrix schenckii* [3], but molecular analyses have reshaped this taxonomical genus, and nowadays we recognize at least four species as causative agents of sporotrichosis: *S. schenckii*, *Sporothrix brasiliensis*, *Sporothrix globosa*, and *Sporothrix luriei* [4,5]. Despite being an infectious disease described more than a century ago [3], it is still considered a neglected infection whose incidence is currently not compulsory to notify to any National Health Ministry [6]. As a consequence, our current knowledge of this disease and its causative agents is relatively limited when compared with candidiasis, cryptococcosis, and aspergillosis, better-studied diseases associated with higher mortality and morbidity rates than sporotrichosis [7]. Nevertheless, the threat posed by sporotrichosis and *Sporothrix* spp. to human beings and animal populations has been brought attention to in recent years. An example of this was provided in 2015 when compared the cumulative amount of published papers dealing with *Sporothrix* or sporotrichosis until 2014, which was 1124, a small number if compared with 69,762 or 46,121 papers published on *Candida* or candidiasis and *Aspeguillus* or aspergillosis, respectively. When this exercise is repeated nowadays with the PubMed database from NCBI (https://pubmed.ncbi.nlm.nih.gov/, accessed on 21 July 2022) there are a total of 3074 manuscripts retrieved after searching the term “*Sporothrix* or sporotrichosis”, which represents an increment of 1950 papers comparing with the amount of 2014. In 2018, we opened the first Special Issue on *Sporothrix* and sporotrichosis in the Journal of Fungi [8], and several outstanding manuscripts communicating original research and authoritative reviews were published (https://www.mdpi.com/journal/jof/special_issues/sporothrix, accessed on 22 July 2022). From 2018 to date, 505 papers have been published on the same subject, representing the 16.4 % of the total literature deposited in PubMed database. This significant accumulation of knowledge on *Sporothrix* and sporotrichosis in the last four years highlights that the research in this area is attracting more interest within the medical mycology community and that this disease is finally gaining momentum. This is why we considered it relevant to open a second Special Issue on *Sporothrix* and sporotrichosis, hoping to underline the recent progress in this area and the new pathways the specialized groups are following to understand this disease and the fungal biology of the *Sporothrix* pathogenic clade members.

Despite globalization allowing us to be connected virtually with any part of the world and to travel long distances in relatively short periods, *Sporothrix* species are not equally distributed worldwide. Instead, some regionalisms are observed: *S. brasiliensis* is found in Brazil and Argentina [9], and *S. globosa* predominantly in Asia, while *S. schenckii* is worldwide distributed [2]. Despite the fact that we know epidemic areas of sporotrichosis in the Americas, the prevalence of the disease in the whole continent is unknown. Therefore, Hernández-Castro et al. performed a bibliographic search for papers published on the subject of *Sporothrix* or sporotrichosis deposited in different bibliographic repositories and published in the last 10 years [10]. They found a total of 124 articles that grouped 12,568 patients, with distribution mainly in South America (87.38%), followed by North America (11.62%), and Central America and the Caribbean (1.00%) [10]. Brazil, Peru, and Mexico were the leading countries in terms of case numbers [10], correlating with already known hyperepidemic areas [1]. Lymphocutaneous infection was the most frequent clinical form, and traditional culture was the main diagnosis method [10].

*S. brasiliensis* has been related to zoonotic outbreaks where the fungal disease is transmitted by cats and the etiological agent is a clonal population [11]. Here, Boechat et al. analyzed 119 *Sporothrix* isolates collected from cats with clinical manifestations of sporotrichosis between 1998 and 2018, in Rio de Janeiro, Brazil [12]. The molecular identification at the species level was performed by T3B PCR fingerprinting and confirmed that all the isolates were *S. brasiliensis* and 31.3% of them showed low intraspecific variation [12]. The interval between lesion onset and first medical visit at the veterinary center, as well as treatment duration until clinical cure, were longer in animals received and treated during the first decade of the epizootic outbreak [12], suggesting that a lack of knowledge and awareness about this disease in the pet owners could be behind this observation and indirectly contributed to spreading the disease. Without a doubt, Rio de Janeiro and other Brazilian areas are hot spots for sporotrichosis, as both zoonotic and epizootic outbreaks of this disease have been reported for at least two decades [13]. The attention has been mainly focused on clinical and veterinary cases, on improving diagnosis and treatments, and the most relevant studies are focused on strategies designed to break animal-animal or animal-human transmission. However, few efforts have been carried out to understand the ecological niche of *Sporothrix* spp. in this geographical area [14]. Here, Almeida-Silva et al. reported the isolation of 18 environmental samples from the hyperepidemic area of Rio de Janeiro, in particular from rural areas with a low cat population density, and were not capable to isolate any fungal colony with morphology similar to *Sporothrix* spp. [14]. However, molecular strategies such as nested-PCR and the species-specific PCR for *S. brasiliensis* were positive for 18 and 5 samples, respectively [14]. Further characterization of amplicons generated by the nested-PCR indicated that were false positive due to amplification of other Ophiostomatales DNA, but three of the five amplicons produced by species-specific PCR were indeed from *S. brasiliensis* [14]. These results indicated that this organism has its ecological niche as other *Sporothrix* species and that molecular techniques such as the ones used in this study lack value if not complemented with DNA sequencing [14].

The most frequent clinical form of human sporotrichosis is the cutaneous-lymphangitic infection, which accounts for 65–85% of all reports, and usually affects upper limbs and, to a lesser extent, lower limbs, shoulders, and the face [1,15]. Systemic infections, such as pulmonary sporotrichosis, are rare and usually are established in immunosuppressed patients [1]. Fichman et al. reported here a 22-year, retrospective cohort study on pulmonary sporotrichosis carried out in the Instituto Nacional de Infectologia Evandro Chagas (Rio de Janeiro, Brazil), and found that during the analyzed period, only 14 patients fulfilled all the inclusion criteria (all of which were diagnosed with an *S. brasiliensis*-caused infection) [16]. This is a surprising figure considering that the clinical center is placed in the heart of a sporotrichosis hyperepidemic area. Primary pulmonary sporotrichosis was observed only in 7.1% of patients, whilst the rest were associated with disseminated infection [16]. Human immunodeficiency virus infection, alcoholism, and chronic obstructive pulmonary disease were the main comorbidities in these patients. It is noteworthy to mention that the mortality rate was 42.9%, and 35.7% of patients were cured [16]. A similar retrospective study conducted in the same National Institute with data collected from 1998 to 2018 looking for patients with disseminated sporotrichosis and treated with amphotericin B is also included in this Special Issue [17]. The study included 73 patients and, as reported in other studies, human immunodeficiency virus infection was the main comorbidity [15,17]. Median doses of amphotericin B were 750 mg and 4500 mg for deoxycholate and lipid complex formulations, respectively, and 52.1%s of patients were cured [17]. The sporotrichosis-associated mortality was observed in 21.9%, while mortality by other causes was reported in 9.6% of patients [17]. It was concluded from the study that infection severity might predict the success of amphotericin B [17]. Another study, but this set in a different brazilian sporotrichosis epidemic area (Rio Grande do Sul state) aimed to analyze the treatment of 28 cases of *S. brasiliensis*-caused cutaneous sporotrichosis [18]. Despite the fact that isolates were suceptible to itraconazole in vitro, 42% of cases did not show clinical response when treated with itraconazole 100 mg per day, requiring changing of doses or combination with other antifungal drugs [18]. Clinical cures were observed after a mean of 187 days of treatment and were dependent on the sporotrichosis clinical form of the patient’s age [18]. Finally, Falcão et al. reported here that the hyperepidemic area of sporotrichosis in Rio de Janeiro state, which was considered a belt along the limits between the capital city and its outskirts, is far from being reduced and under control [19]. They report a progressive expansion of the affected area, even though local public health measures have been applied since 2011 [19].

Thus far, most of the basic and biological aspects of members of the *Sporothrix* genus have been studied in *S. schenckii*, and the cell wall structure and composition are not an exception [20,21,22]. One particular feature of the *S. schenckii* cell wall is the presence of rhamnoconjugates attached to lipids and proteins, the latter named peptidorhamnomannan [23]. The presence of rhamnose-containing molecules in fungal cells is not common, and despite several rhamnosyltransferases having been isolated and characterized in bacteria, so far no fungal enzyme with this activity has been isolated yet [24]. Here, using a hidden Markov model profile strategy, Mora-Montes et al. reported the identification of five putative rhamnosyltransferases encoding genes within the *S. schenckii* genome [25]. Two of them were found to be expressed in yeast-like cells and during interaction with the host. To demonstrate their enzyme activity, the coding regions were heterologously expressed in *Escherichia coli*, the recombinant proteins purified, and the enzyme activity characterized [25]. Both proteins, named Rht1 and Rht2, showed UDP-rhamnose-dependent rhamnosyltransferase activity and were capable of transferring up to two rhamnose units to a mannobiose acceptor, generating rhamnomannan *in vitro* [25]. The study of the *Sporothrix* cell wall is also relevant since it is the fungal structure that enters in contact with the host immunity in the first place and this interaction triggers a series of events that could lead to a protective anti-*Sporothrix* immune response or the pathogen dissemination [26,27]. Currently, there is scarce information about *Sporothrix*-human keratinocytes interaction. Here, Paredes-Rojas et al. explored this relevant aspect using *S. schenckii* yeast-like cells and conidia and the human keratinocyte cell line HaCaT [28]. The main observations included that fungal cells induced alterations of the actin cytoskeleton, formation of membrane protuberances, loss of stress fibers, and overexpression of the surface receptors MR, TLR6, CR3, and TLR2 [28]. Moreover, both conidia and yeast-like cells showed similar abilities to stimulate pro-inflammatory and anti-inflammatory cytokines, expecting for the case of TNF-α, whose production was significant only when the cell line was stimulated with conidia [28]. Another important function of the fungal cell wall is to serve as a molecular scaffold to display molecules that can contribute in pathogenesis and virulence [29,30]. Here, García-Carnero and Martínez-Álvarez provide an up-to-date review paper on the most recent information related with virulence factors already rerpoted in *S. schenckii* [31]. These include cell wall adhesins, melanin production, extracellular and intracellular proteinases, extracellular vesicles, and biofilm formation [31]. As part of the adhesins repertoire found on the *S. schenckii* cell wall, García-Carnero et al. showed that the cell wall peptidorhamnomannan is composed of a heterogeneous mix of different proteins, and among them Hsp60 and Pap1 are the most abundant ones [32]. Recombinant versions of both proteins had adhesive properties to different extracellular matrix components and were capable of inducing immunological priming when injected into laboratory animals, and this protected them from a fatal inoculum of *S. schenckii* yeast-like cells [32]. In line with these observations, fungal cells treated with specific anti-Pap1 or anti-Hsp60 antibodies were not capable of killing laboratory animals, suggesting that both proteins participate in the host-fungus interaction [32].
